# Structured Multidisciplinary Follow-Up After Pediatric Intensive Care: A Model for Continuous Data-Driven Health Care Innovation

**DOI:** 10.1097/PCC.0000000000003213

**Published:** 2023-02-17

**Authors:** Eleonore S. V. de Sonnaville, Job B. M. van Woensel, Johannes B. van Goudoever, Marieke H. Otten, Lorynn Teela, Cornelieke S. H. Aarnoudse-Moens, Suzanne W. J. Terheggen-Lagro, Annelies E. van der Hulst, Marc Engelen, Marsh Kӧnigs, Jaap Oosterlaan, Hennie Knoester

**Affiliations:** 1 Amsterdam UMC, University of Amsterdam, Emma Children’s Hospital, Department of Pediatric Intensive Care, Amsterdam Reproduction and Development Research Institute, Meibergdreef 9, Amsterdam, The Netherlands.; 2 Amsterdam UMC, University of Amsterdam, Emma Children’s Hospital, Department of Pediatrics, Emma Children’s Hospital Amsterdam UMC Follow Me Program & Emma Neuroscience Group, Amsterdam Reproduction and Development Research Institute, Meibergdreef 9, Amsterdam, The Netherlands.; 3 Amsterdam UMC, University of Amsterdam & Vrije Universiteit, Emma Children’s Hospital, Department of Pediatrics, Amsterdam Reproduction and Development Research Institute, Meibergdreef 9, Amsterdam, The Netherlands.; 4 Amsterdam UMC, University of Amsterdam, Emma Children’s Hospital, Department of Child and Adolescent Psychiatry & Psychosocial Care, Amsterdam Reproduction and Development & Amsterdam Public Health Research Institutes, Meibergdreef 9, Amsterdam, The Netherlands.; 5 Amsterdam UMC, University of Amsterdam, Emma Children’s Hospital, Department of Pediatric Pulmonology and Allergy, Amsterdam Reproduction and Development & Infection and Immunity Research Institutes, Meibergdreef 9, Amsterdam, The Netherlands.; 6 Amsterdam UMC, University of Amsterdam, Emma Children’s Hospital, Department of Pediatric Cardiology, Meibergdreef 9, Amsterdam, The Netherlands.; 7 Amsterdam UMC, University of Amsterdam, Emma Children’s Hospital, Department of Pediatric Neurology, Amsterdam Leukodystrophy Center, Amsterdam Neuroscience & Amsterdam Gastroenterology Endocrinology Metabolism Research Institutes, Meibergdreef 9, Amsterdam, The Netherlands.

**Keywords:** child, follow-up studies, pediatric intensive care units, postintensive care syndrome

## Abstract

**OBJECTIVES:**

Morbidity after PICU admission for critical illness is a growing concern. Sequelae may occur in various domains of functioning and can only appropriately be determined through structured follow-up. Here, we describe the process of designing and implementing a structured multidisciplinary follow-up program for patients and their parents after PICU admission and show the first results illustrating the significance of our program.

**DESIGN:**

Prospective observational cohort study.

**SETTING:**

Outpatient PICU follow-up clinic.

**PATIENTS:**

Patients 0–18 years old admitted to our PICU.

**INTERVENTIONS:**

None.

**MEASUREMENTS AND MAIN RESULTS:**

In our structured multidisciplinary follow-up program, follow-up care is provided by a pediatric intensivist and psychologist and in addition, depending on patient’s critical illness and received PICU treatment(s), by a pediatric pulmonologist, cardiologist, neurologist, and/or neuropsychologist. All consultations are scheduled consecutively. Collected data are stored in a hospital-wide data warehouse and used for yearly health care evaluation sessions as well as scientific research. Challenges in organizing this follow-up program include technological challenges, providing time-efficient care, participation rate, and completeness of questionnaires. In our experience, a dedicated team is essential to tackle these challenges. Our first results, obtained in 307 of 388 referred patients (79.1%), showed the diversity of problems arising after PICU discharge, including physical, neurocognitive, and psychosocial sequelae. In addition, our data also reflected the risk of psychosocial problems among parents. Within the limited operation time of our follow-up program, the program has evolved based on our experiences and the data collected.

**CONCLUSIONS:**

We successfully developed and implemented a structured multidisciplinary follow-up program for patients and their parents after PICU admission. This program may help to timely initiate appropriate interventions, improve the standard of care during and after PICU admission, and facilitate scientific research on outcome and prognosis after PICU admission.

**RESEARCH IN CONTEXT**

- Morbidity after PICU admission is a growing concern. Given the distinct heterogeneity of the PICU population and potential consequences in a wide range of outcome domains, the prognosis after PICU admission is uncertain, in turn challenging clinical follow-up.
- Few PICU follow-up programs exist, which vary in terms of the included patients, involved health care professionals, follow-up moments, and/or assessed outcomes. Moreover, most PICU follow-up programs lack structured data collection, which is essential for health care evaluation and scientific research.
- Structured multidisciplinary follow-up, including structured data collection, can importantly contribute to improve outcomes of individual patients, improve the standard of care during and after PICU admission, and facilitate scientific research on outcome and prognosis of patients after PICU admission.

**WHAT THIS STUDY MEANS**

- Given the distinct heterogeneity of the PICU population and wide range of potential impairments in functioning, we propose that structured multidisciplinary follow-up is required for patients and their parents after PICU admission.
- Our structured multidisciplinary follow-up program, including structured data collection, serves as an example of how clinical care, health care evaluation, and scientific research can be integrated to continuously fuel a cycle of care innovation.
- The accumulating outcome data will ultimately provide better insight in disease- and treatment-specific patient outcomes, thereby facilitating targeted follow-up of high-risk patients and allowing further tailoring of the content of follow-up offered to those domains of functioning threatened.

With advances in pediatric intensive care, the mortality of children admitted to the PICU has decreased dramatically during the past decades (1, 2). As a consequence, morbidity after PICU admission is a growing concern, recently acknowledged as the pediatric postintensive care syndrome (3–7). Sequelae are described in physical, neurocognitive, and psychosocial functioning (3–7) and vary between and within the heterogeneous patient groups that are admitted to the PICU (3). In addition, admission of a child to the PICU is a major life event for the child’s family and may impact family functioning, acknowledged as the postintensive care syndrome-family (8, 9).

Current literature on sequelae after PICU admission is fragmented, mainly comprising small-sized cross-sectional studies often focusing on a specific patient group and/or a limited set of outcomes (10). Given the distinct heterogeneity of the PICU population and potential consequences in a wide range of outcome domains, the prognosis after PICU admission is uncertain, in turn challenging clinical follow-up. Currently, a few descriptions of PICU follow-up programs have emerged in the literature (11–17). To our knowledge, no standardized structure for follow-up care after PICU admission exists. The few existing programs described in the literature vary in terms of the included patients (e.g., focused on patients with neurologic diagnoses), involved health care professionals (e.g., physician only), follow-up moment(s), and/or assessed outcomes. Moreover, most PICU follow-up programs lack structured data collection (11), which is essential for health care evaluation and scientific research on outcome and prognosis of patients after PICU admission.

In order to gain more insight in the presence or absence of sequelae in the various domains of functioning in different patient groups and to improve outcomes of patients after PICU admission, we propose that there is a need of structured multidisciplinary follow-up for patients and their parents after PICU admission. Structured follow-up and collection of outcome data within a multidisciplinary follow-up program can importantly contribute: 1) to improve outcomes of individual patients by facilitating timely support and appropriate intervention, if required; 2) to improve the standard of care during and after PICU admission by providing insight in extent and severity of sequelae; and 3) to facilitate scientific research on outcome and prognosis of patients after PICU admission. The objective of this article is to describe the design and implementation of a structured multidisciplinary follow-up program for patients and their parents after PICU admission in our hospital and to show the first results obtained in patients and their parents to illustrate the significance of our program. Our program integrates clinical care, health care evaluation, and scientific research to fuel a cycle of care innovation.

## METHODS

### Setting and Patients

Our multidisciplinary follow-up program is provided to patients 0–18 years old admitted to the PICU of the Emma Children’s Hospital of the Amsterdam University Medical Centers (UMCs), The Netherlands, a tertiary 14-bed PICU, serving the greater Amsterdam area. Detailed information on the inclusion and exclusion criteria for follow-up care is provided in **Table 1**. Most patients referred to the program are previously healthy children with unplanned PICU admission (> 24 hr), not receiving similar follow-up elsewhere. The PICU follow-up program is part of hospital’s follow-up program (Follow Me program) that aims to offer high-quality, evidence-based, multidisciplinary follow-up to all level three pediatric patients, improve the standard of care, and facilitate scientific research on outcome and prognosis.

**TABLE 1.** Inclusion and Exclusion Criteria for Follow-Up After Admission to the PICU and Involved Health Care Professionals

Inclusion and Exclusion Criteria for Follow-Up	Pediatric Intensivist	Pediatric Psychologist	Pediatric Pulmonologist	Pediatric Cardiologist	Pediatric Neurologist	Pediatric Neuropsychologist
Inclusion criteria (irrespective of length of PICU stay)						
Trauma other than traumatic brain injury	X	X				
Sepsis	X	X			X	X
Meningitis	X	X			X	X
Traumatic brain injury	X	X			X	X
Multisystem Inflammatory Syndrome-Children and/or COVID-19	X	X			X	X
Cardiopulmonary resuscitation	X	X			X	X
Extracorporeal membrane oxygenation	X	X			X	X
Inclusion criteria (PICU stay ≥ 24 hr)						
Previously healthy and unplanned PICU admission	X	X				
Central venous catheter	X	X				
Traumatic intubation	X	X				
Delirium	X	X			X	X
Status epilepticus	X	X			X	X
Inclusion criteria (PICU stay ≥ 48 hr)						
Bronchiolitis	X	X	X			
≥ 48 hr sedatives/anesthetics	X	X			X	X
(Septic) shock with ≥ 48 hr inotropic agents	X	X		X	X	X
Acute kidney injury during PICU admission^a^	X	X				
≥ 72 hr (non)invasive mechanical ventilation	X	X	X			
Exclusion criteria	Receives similar follow-up care elsewhere (e.g., diabetic ketoacidosis, supraventricular tachycardia, chronic mechanical ventilation, palliative care, severe neurocognitive, and/or motor problems requiring admission to a rehabilitation center). Developmental disorder or social situation precludes follow-up.					
Additional exclusion criterion for pediatric neuropsychologist	Developmental disorders known to impact on neurocognitive development; physical conditions and/or behavioral deficits interfering with the ability to adequately perform neurocognitive testing.					

a Creatinine elevation > 25% and/or diuresis < 0.5 mL/kg/hr for ≥ 8 hr without known kidney disease.

### Preparing Phase

Prior to implementation of the follow-up program, follow-up protocols were developed based on international (6) and national guidelines (18), consensus statements, systematic reviews, other empirical literature, and clinical experience (in order of preference). All protocols were reviewed and approved after several consensus meetings by all members of the follow-up program. A dedicated team was constituted, including a director, project manager, pediatric intensivists, protocol holders (involved health care professionals, i.e., the pediatric psychologist, pediatric pulmonologist, pediatric cardiologist, pediatric neurologist, and pediatric neuropsychologist), case managers, a parent counselor, and a PhD student. The follow-up program has been implemented since March 2018.

### Structure of the Follow-Up Program

The follow-up program provides comprehensive and structured follow-up care, tailored to the child’s critical illness and received PICU treatment(s). The follow-up consultation always comprises consultation with both a pediatric intensivist and a case manager and a separate consultation with a psychologist (one single psychologist delivers consultations to both patients and parents). Depending on the patient’s critical illness and received PICU treatment(s), patients additionally may receive care by the pediatric pulmonologist, pediatric cardiologist, pediatric neurologist, and/or pediatric neuropsychologist. Detailed information on the health care professionals involved is provided in Table 1.

The involved case managers are certified PICU nurses who have rotations at the PICU and work as case manager for the follow-up program 1 day per week. They are the main link between the patient, their parents, and the health care professionals involved in the follow-up program. The case manager informs families on the follow-up program during PICU admission and interfaces with families during consultations. Furthermore, the case manager is responsible for coordinating appointments with different subspecialists.

The first follow-up consultation takes place 3–6 months after PICU discharge. We identified the 3- to 6-month follow-up time frame, because mild problems—not requiring evaluation by the health care professionals—have already resolved spontaneously by that time. For example, the rate of child and parent posttraumatic stress symptoms has shown a sharp decrease in the first months after pediatric injury (19). Patients and/or parents having concerns prior to consultation are invited to contact the case manager. Our program uses a standardized assessment battery to evaluate neurocognitive function that includes tests appropriate for children 6 years old and above, as by that age the full range of neurocognitive functions can be assessed. Therefore, patients younger than 6 years at first follow-up who meet the inclusion criteria for consultation by the pediatric neurologist and neuropsychologist (including neurocognitive screening) will return for follow-up at the age of 6 years. Furthermore, in case of prominent neurocognitive and/or motor problems determined during PICU admission or at the first outpatient visit 3–6 months after PICU discharge, children will be directly referred for neurocognitive assessment and support, either in our hospital or in a rehabilitation center. Patients also return for follow-up at the age of 6 for consultation by the pediatric pulmonologist, as from that age lung function testing by spirometry can be performed and it is possible to officially diagnose asthma (20). In addition, the pediatric pulmonologist and the pediatric rehabilitation physician are always present during the multidisciplinary team meeting, and in of case pulmonary problems and/or motor problems, an earlier appointment is scheduled with the pediatric pulmonologist and/or pediatric rehabilitation physician, respectively.

Since the COVID-19 pandemic, visits to the outpatient clinics were discouraged; patients and parents were therefore first invited for video consultation. If deemed necessary, patients and parents were then invited to the outpatient clinic. Most parents prefer video consultation, as they do not have to travel and consultations are easier to arrange. Therefore, we decided to continue video consultation. In practice, this means that patients who are only scheduled for consultation by the pediatric intensivist and psychologist (majority of the patients) receive video consultation and are only invited to the outpatient clinic if further evaluation is required. Patients who are scheduled for consultation by the pediatric pulmonologist, pediatric cardiologist, pediatric neurologist, and/or pediatric neuropsychologist are always invited to the outpatient clinic.

Consultation time of involved health care professionals is approximately 30 minutes per consultation, spirometry takes approximately 30–60 minutes, and neurocognitive screening takes approximately 2.5 hours. All consultations are scheduled consecutively in the interest of time-efficiency for patient and parents. In addition, this facilitates multidisciplinary consultations between the health care professionals involved. After the consultations have taken place, a multidisciplinary team meeting is scheduled, in which all monitored outcomes are shared and evaluated and recommendations for further evaluation and treatment are discussed. Parents are approached by phone by the pediatric intensivist or psychologist (depending on the observed problems) to explain recommendations discussed in the multidisciplinary team meeting. If necessary, further follow-up appointments are scheduled or referrals are made. In case patients and/or parents need additional support, this will be either provided by the health care professional who performed consultation (e.g., the psychologist, pediatric pulmonologist) or patients are referred to other health care professionals (e.g., the ear nose and throat physician).

Clinician-reported outcomes are registered in discrete registrations and automatically appear in the standardized written report, which is sent to the general practitioner and other treating physicians and subsequently stored in the patient’s electronic medical record. Health care professionals participating in the follow-up program are responsible for entry of all medical information in the electronic medical files. This is facilitated by the use of precoded answer options.

### Data Collection and Outcome Measures

Patients (from the age of 8 yr) and/or their parents (of patients 0–18 yr old) complete questionnaires online before consultation via a web-based portal (KLIK Patient Reported Outcome Measures [PROM] portal; https://www.hetklikt.nu). Questionnaires pertain to physical and psychosocial functioning (**eTable 1**, http://links.lww.com/PCC/C336) and are used to prepare the consultation and focus the consultation on the specific issues reported (21). The physical outcomes are discussed by the pediatric intensivist, and the psychosocial outcomes are discussed by the psychologist during the outpatient consultation (both qualified to interpret the results). For some validated questionnaires, a license is required. The KLIK PROM portal has the necessary licenses (eTable 1, http://links.lww.com/PCC/C336).

During the consultations, various outcomes are evaluated, including generic somatic functioning, specific organ functioning, psychosocial and neurodevelopmental functioning, and parental psychosocial well-being. Outcomes are registered in discrete registrations. Each outcome of interest is covered by descriptions (items) in the electronic patient file describing the exact information to be gathered from history taking and physical examination. Some of these items offer follow-up items, depending on the response chosen. **eTable 2** (http://links.lww.com/PCC/C336) displays specific outcome measures assessed by the involved health care professionals, including key references underpinning the importance of involved health care professionals and assessed outcomes.

### Database and Data Warehouse

Data gathered with the patient- and parent-reported online questionnaires are stored in a local data repository, and clinician-reported outcomes are registered in discrete registrations and stored in the electronic patient record (Epic Hyperspace, Verona, WI). All data from the electronic patient records collected during PICU admission and during follow-up (eTable 2, http://links.lww.com/PCC/C336) are stored in a hospital-wide data warehouse and extracted in a standardized format via an SQL server. The extracted data are visualized in a real-time available dashboard built in Microsoft PowerBI (Microsoft Corporation, Redmond, WA) (**eFig. 1**, http://links.lww.com/PCC/C337; legend, http://links.lww.com/PCC/C336). The databases are available for routine outcome monitoring and research purposes by the involved caregivers and members of the supporting hospital’s follow-up program team, if parents and patients from the age of 12 provided informed consent for use of the data collected during PICU admission and during follow-up. The Amsterdam UMC medical ethical committee approved the use of these data for routine outcome monitoring and research purposes (W21_516 no. 21.569).

### Health Care Evaluation

**Figure 1.**
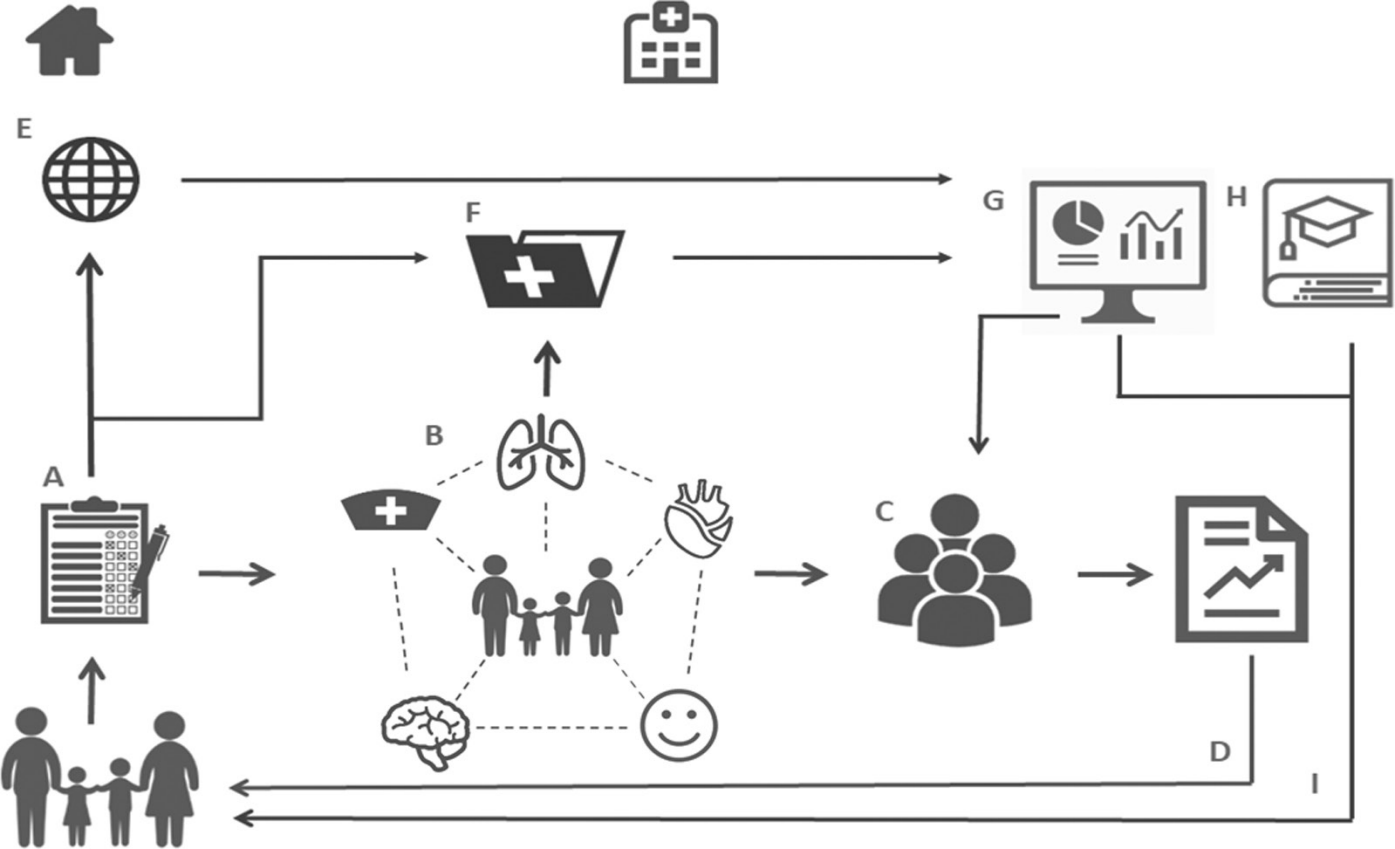
Overview of data collection of patient outcomes and the use of these data for health care evaluation and scientific research. **A**, Patients and parents complete online questionnaires; **B**, consultations with the assigned health care professionals, with discrete registration of outcomes; **C**, multidisciplinary team meeting; **D**, standardized written report is sent to the general practitioner and other treating physicians. All collected data are stored in the local data repository (**E**) or in the electronic patient’s record (**F**) and extracted for the visualization of process and outcome indicators in a dashboard (**G**) used for health care evaluation and scientific research (**H**), which in turn contributes to improvements in the standard of care during and after PICU admission (**I**).

Yearly health care evaluation sessions are organized with the multidisciplinary team, representatives of the hospital’s follow-up program and management team. In these sessions, process indicators and all assessed outcomes are evaluated at a population level, with the aim to define targets for improvement in the standard of care during and after PICU admission. Process indicators and outcomes are evaluated using the real-time available dashboards. Evaluation of outcome data may also give rise to adjustments in the follow-up protocols and may inspire research questions. **Figure 1** shows the data flow in the primary care process and evaluation cycle.

## RESULTS

### Patients

We here provide a brief overview of selected outcomes obtained during the follow-up care provided to patients discharged from our PICU between October 2017 and April 2021. Of the 1,752 (unique) patients, 388 patients met inclusion criteria for follow-up. Patients not meeting inclusion criteria had elective admission for postoperative care, received similar follow-up care elsewhere, or passed away. A total of 307 patients (79.1% of the patients eligible for follow-up) visited our follow-up clinic between March 2018 and July 2021. **Table 2** displays the demographic and clinical characteristics of these patients.

**TABLE 2.** Demographic and Clinical Characteristics of the Patients (*N* = 307) Participating in the Follow-Up Program, 3–6 Months After PICU Admission

Demographic and Clinical Characteristics	*n* (%) or Median (IQR)
Male sex, *n* (%)	178 (58.0)
Age at PICU admission (mo), median (IQR)	14.0 (1.0–92.0)
PICU length of stay (d), median (IQR)	4.0 (2.0–7.0)
Mechanical ventilation, *n* (%)	163 (53.1)
Inotropic agents, *n* (%)	46 (15.0)
Cardiopulmonary resuscitation, *n* (%)	13 (4.2)
Extracorporeal membrane oxygenation, *n* (%)	0 (0.0)
Primary PICU admission indication	
Respiratory, *n* (%)	
Upper airway obstruction	30 (9.8)
Asthma	33 (10.7)
Bronchiolitis	78 (25.4)
Respiratory insufficiency other than asthma/bronchiolitis	19 (6.2)
Circulatory, *n* (%)	
Asystole	9 (2.9)
Shock (anaphylactic/cardiogenichypovolemic/septic)	24 (7.8)
Multisystem InflammatorySyndrome-Children	15 (4.9)
Neurologic, *n* (%)	
Nontraumatic intracranial hemorrhage	4 (1.3)
Meningitis/encephalitis/empyemaabscess	13 (4.2)
Status epilepticus	17 (5.5)
Trauma, *n* (%)	
Abdominal trauma	11 (3.6)
Traumatic brain injury with/withoutother trauma	22 (7.2)
Other trauma	2 (0.7)
Congenital malformation, *n* (%)	
Congenital gastrointestinal malformation	10 (3.3)
Congenital cor vitium	1 (0.3)
Miscellaneous, *n* (%)	19 (6.2)

IQR = interquartile range.

### Outcomes

**Figure 2.**
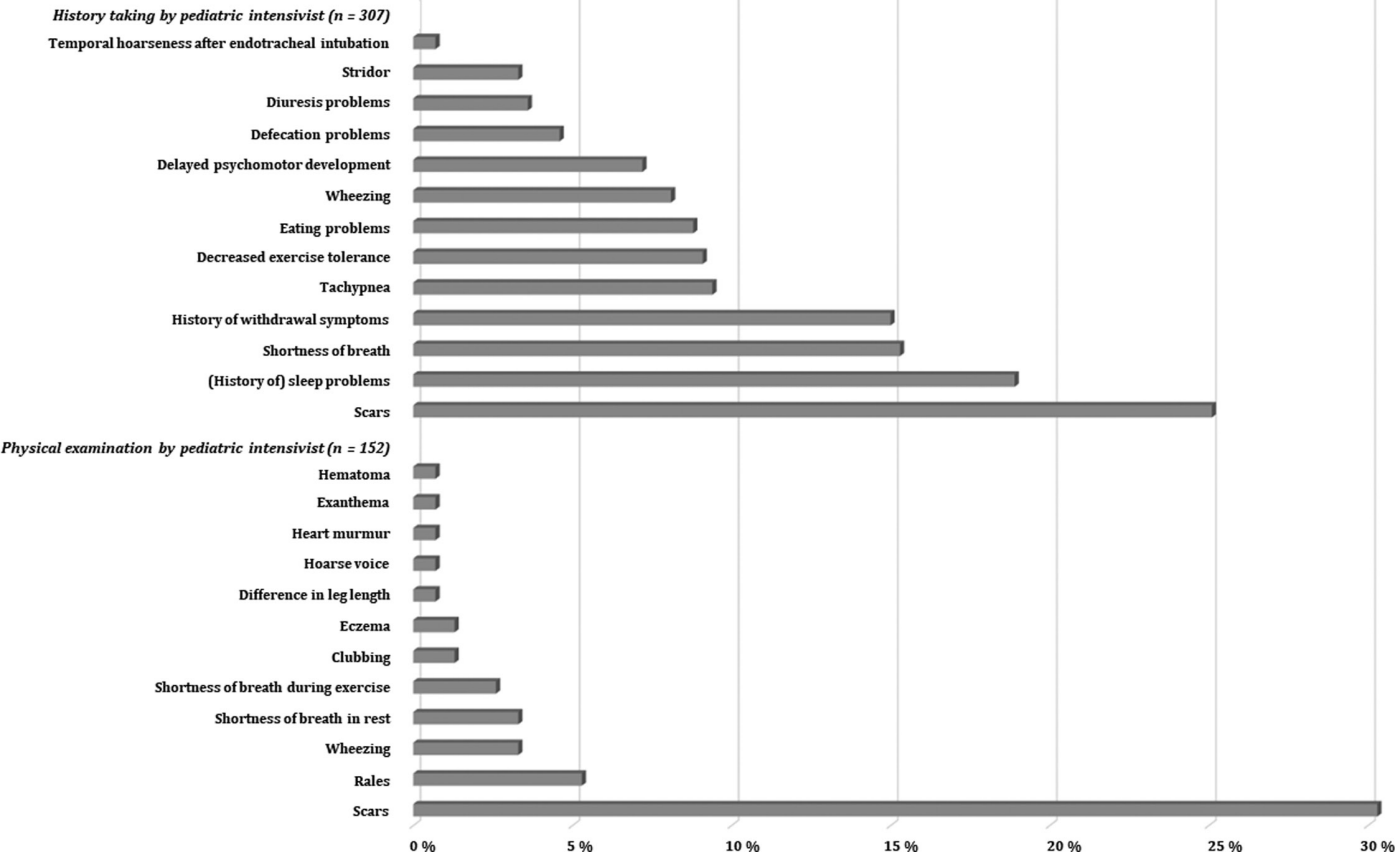
Physical outcomes revealed during history taking (*n* = 307) and physical examination (*n* = 152) by the pediatric intensivist at the outpatient follow-up clinic at 3–6 mo after PICU discharge. The pediatric intensivist evaluates whether the outcomes are normal according to patients’ age. In case of sleep problems, parents reported their child to have the following problems: problems falling asleep (43.1%), nightmares (17.2%), not wanting to sleep alone (34.5%), sleeps too short (13.8%), and wakes up during the night (41.4%). The majority of the scars (94.8%) had their origin in the period of PICU admission (84.4% due to PICU treatment and 13.0% due to the disease that was reason for PICU admission).

**Figure 2** displays the physical outcomes obtained by the pediatric intensivist. History taking by the pediatric intensivist was performed in all 307 patients, and physical examination was performed in 152 patients, as since the COVID pandemic patients were only invited to the outpatient clinic if necessary. Most frequently revealed problems during history taking were scars (25.1%), sleep problems (18.9%), and shortness of breath (15.3%). Most frequently revealed problems during physical examination were scars (30.3%), rales (5.3%), and wheezing (3.3%) on lung auscultation.

**Figure 3.**
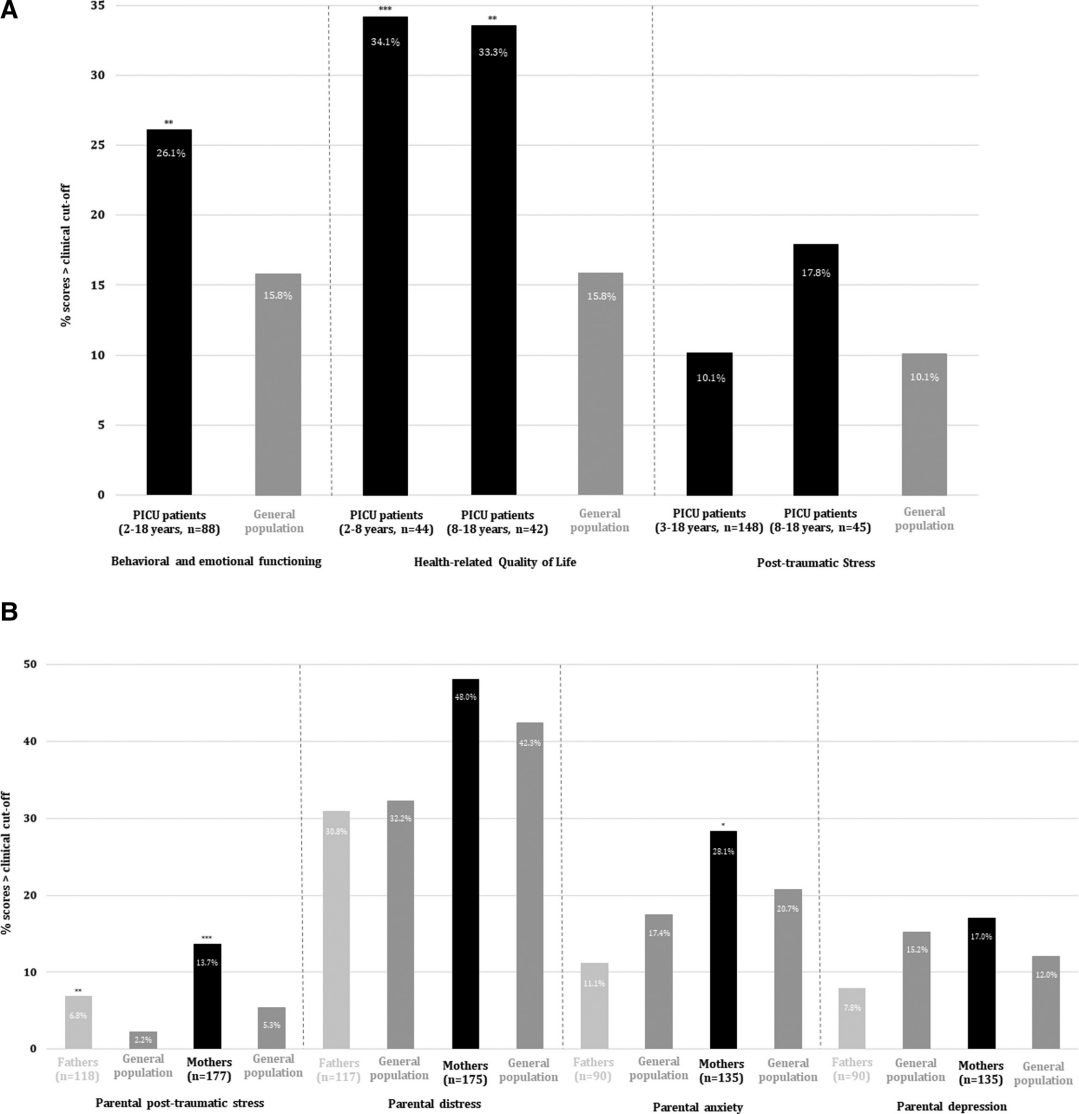
Psychosocial outcomes of PICU patients (**A**) and parents (**B**), compared with the general population. *p < 0.05. **p < 0.01. ***p < 0.001. “Behavioral and emotional functioning” assessed by the parent-reported Strengths and Difficulties Questionnaire (*n* = 88). Higher scores indicate greater severity of problems. Percentage of patients with a total score greater than 1 sd and compared with the general population (22). “Health-related Quality of Life” assessed by the Pediatric Quality of Life Inventory (2–8 yr parent-reported, *n* = 44 and 8–18 yr patient-reported, *n* = 42). Higher scores indicate better health-related quality of life. Percentage of patients with a total score less than –1 sd and compared with the general population (23, 24). “Post-traumatic stress” assessed by the Children’s Revised Impact of Event Scale (CRIES-13, 3–18 yr parent-reported, *n* = 148 and 8–18 yr patient-reported, *n* = 45). Higher scores indicate greater severity of problems. Percentage of patients with “probable posttraumatic stress disorder (PTSD)” defined as a total score of greater than or equal to 31 (parent-reported) or greater than or equal to 30 (patient-reported). Patient-reported outcomes compared with the general population (25). There are no normative data available for the parent-reported CRIES-13. “Parental PTSD” assessed until February 2021 by the Self-Rating Scale for Posttraumatic Stress Disorders (SRS-PTSD, assessing *Diagnostic and Statistical Manual of Mental Disorders* [DSM]-IV symptoms of PTSD, *n* = 226) and since February 2021 by the PTSD Checklist for DSM-V (PCL-5, assessing DSM-V symptoms of PTSD, *n* = 69). Higher scores represent more posttraumatic stress symptoms. For the SRS-PTSD, a diagnosis of PTSD is considered likely if one “intrusions” symptom, three “avoidance” symptoms, and two “hyperarousal” symptoms are present. For the PCL-5, a diagnosis of PTSD is considered likely if one “intrusions” symptom, one “avoidance” symptom, two “negative alterations in cognitions and mood” symptoms, and two “arousal and reactivity” symptoms are present. Percentage of parents for whom the diagnosis of PTSD is considered likely and compared with the general population for the SRS-PTSD (26) only as no normative values are available for the PCL-5. “Parental distress” assessed by the Distress Thermometer for Parents (*n* = 292). Higher scores indicate greater severity of distress. Percentage of parents with clinically elevated distress based on a score of greater than or equal to 4 (27) and compared with the general population (27). “Parental anxiety and depression” assessed by the Hospital Anxiety and Depression Scale (*n* = 225). Higher scores indicate greater severity of anxiety and depression. Percentage of parents with clinically significant anxiety and depression based on a scale score of greater than or equal to 8 and compared with the general population (28).

**Figure 3** displays the psychosocial outcomes of patients and parents. Percentages of patients and parents obtaining clinical scores (i.e., scores above cutoff scores defining scores requiring further assessment) were compared with the prevalence observed in the general population. The prevalence of clinical scores with respect to behavioral and emotional functioning and health-related quality of life was higher in patients than observed in the general population. The prevalence of clinical scores for fathers and mothers with respect to parental posttraumatic stress and for mothers with respect to parental anxiety was higher than the prevalence observed in the general population.

Evaluation by the pediatric pulmonologist took place in only four patients, as the great majority of patients was too young (younger than 6 yr) to perform lung function testing. Two of four patients had complaints during follow-up: one patient had chronic productive cough, and another patient had wheezing complaints during follow-up and received additional follow-up.

Evaluation by the pediatric cardiologist took place in nine patients. Only mild to moderate problems were identified including moderate exercise tolerance (11.1%), shortness of breath (11.1%), and mild tachypnea (33.3%). Electrocardiogram and echocardiogram identified mild hypertrophy of the septum and right ventricle in one patient, which normalized spontaneously during additional follow-up. Electrocardiogram and echocardiogram revealed no abnormalities in all other patients.

The pediatric neurologist evaluated 27 patients and identified small-fiber polyneuropathy in one patient who was admitted for Multisystem Inflammatory Syndrome-Children (MIS-C). In all other patients, no (new) neurologic findings were identified.

Neurocognitive testing was performed in 43 patients. Mean estimated full-scale intelligence quotient (FSIQ) was 98.7 (sd 15.5, range 63.0–131.0). In total, 18.6% of the patients (8/43) had a FSIQ less than –1 sd (i.e., < 85), which is not significantly different compared to normative data (*p* = 0.61).

## DISCUSSION

This article presents the design and implementation of a structured multidisciplinary follow-up program for patients and their parents after PICU admission, including the first results obtained in patients and their parents to illustrate the significance of our program. To our knowledge, no standardized structure for follow-up care after PICU admission exists. Current PICU follow-up programs (11–17) vary widely with respect to, among others, the included patients, involved health care professionals, follow-up moment(s), and assessed outcomes. Moreover, most PICU follow-up programs lack structured data collection (11), which is essential for health care evaluation and scientific research on outcome and prognosis of critically ill children. Defining features of our structured multidisciplinary PICU follow-up program include the following: 1) a multidisciplinary team that is responsible for patient follow-up, 2) structured follow-up using evidence-based protocols and well-defined inclusion and exclusion criteria for follow-up, 3) the use of a one stop shop format for consultations, with consultations of all health care professionals scheduled consecutively, 4) the use of online questionnaires on physical and psychosocial functioning to prepare for the consultations, 5) structured data collection using discrete registrations, 5) yearly health care evaluation sessions aimed at improving the standard of care during and after PICU admission, and 6) the use of outcome data for scientific research. Our program integrates clinical care, health care evaluation, and scientific research, fueling a cycle of care innovation.

In order to achieve a holistic view on an individual patient’s functioning, our structured follow-up protocols include a comprehensive assessment of important outcomes evaluated by a multidisciplinary team. In addition, psychosocial functioning is also assessed in parents. This approach follows the International Classification of Functioning, Disability and Health model of the World Health Organization (29), which acknowledges that health is a multidimensional construct that is under the influence of personal and environmental factors. Our first results confirm the presence of a diversity of problems arising after PICU admission, including physical, neurocognitive, and psychosocial sequelae. In addition, our data also confirm the risk for psychosocial problems among parents (8, 9). These findings underline the importance of structured multidisciplinary follow-up of patients and their parents after PICU admission.

Within the limited operation time of our follow-up program, the program has evolved based on our experiences and the data collected. For example, since the COVID pandemic, patients who are only scheduled for consultation by the pediatric intensivist and psychologist (majority of the patients) receive video consultation and are only invited to the outpatient clinic if further evaluation is required. As most parents prefer video consultation due to time-efficiency, we decided to continue video consultation. Furthermore, evaluation of the neurologic exams conducted as part of the consultations by the pediatric neurologist identified new neurological abnormalities in only one patient (3.7%), which were also identified by the pediatric intensivist. Therefore, we have removed the neurologic exam from the structured follow-up, which now only takes place in case of concerns raised by the pediatric intensivist. Furthermore, the collected data can be used to evaluate changes in the protocols used at the PICU. For example, before the introduction of our current follow-up program, we studied the outcomes of children unexpectedly admitted to our PICU 3 months after discharge. In that study, seven of 186 children (3.8%) were diagnosed with postthrombotic syndrome (30). This finding has led to the greater awareness of the risk on postthrombotic syndrome and development of a protocol on central venous catheters, including ultrasound in case of suspected problems and prophylactic anticoagulants during PICU admission. Since then, the prevalence of postthrombotic syndrome has dropped to 0% as assessed in the patients who visited our structured multidisciplinary follow-up program.

Until now, only few patients were evaluated by the pediatric cardiologist, pediatric pulmonologist, and pediatric neuropsychologist, as few patients met the inclusion criteria for evaluation by the pediatric cardiologist and most patients were too young to perform lung function and neurocognitive testing at the time of follow-up. Therefore, based on the current results, we cannot evaluate the beneficial value of these subspecialists to our program. Nevertheless, as part of our follow-up program, we studied a cohort of patients now 6–12 years old and previously admitted to the PICU for bronchiolitis requiring invasive mechanical ventilation. The results showed that these patients are at risk of adverse long-term pulmonary and neurocognitive outcomes, highlighting the importance of long-term pulmonary and neurocognitive follow-up after PICU admission (31, 32). All children in this cohort with adverse pulmonary outcomes had obstructive lung function, which can only be detected by spirometry and would thus involve a pediatric pulmonologist (31). The findings of our follow-up program and the existing literature show a great diversity of adverse outcomes after PICU admission and thus call for a multidisciplinary approach to follow-up of PICU patients.

Currently, our program offers neurocognitive follow-up to children 6 years old and older, with younger children receiving delayed neurocognitive follow-up at the age of 6 years. This approach was chosen as at that age, most neurocognitive functions have been fully formed and a wide range of assessment materials is available (33). A limitation of this approach is that children younger than 6 years old suffering from neurocognitive sequelae might remain undetected and are at risk of delayed support. Assessment tools with established clinimetric properties are available to detect the impact of disease on early neurocognitive development. Although these measures often show limited predictive value for future neurocognitive functioning (34), they provide the best available methods to detect those children in need of intervention and may be helpful in guiding the decisions on adequate support. Recently new and promising assessment methods have become available for young children including methods relying on eye-tracking methodology and applied in babies and infants (35, 36) as well as the National Institute of Health Toolbox for the Assessment of Neurological and Behavioral Function (37), suitable for children from 3 years old. As the majority of the PICU population is too young for the current neurocognitive tests 3–6 months after PICU discharge, we consider including neurocognitive tests appropriate for younger children in our program.

A structured approach to multidisciplinary follow-up allows us to create a consecutive cohort, with accumulating patient data, which could be a solution to the challenged literature describing the heterogeneous PICU population with small-sized studies and limited sets of outcomes. Hereby, it allows to create better insight in disease- and treatment-specific outcomes that can contribute to patient education and shared-decision making. Better insight into outcomes will facilitate targeted follow-up of high-risk children and allow further tailoring of the content of follow-up offered to those domains of functioning threatened. The development of personalized prognostic models may importantly facilitate targeted follow-up (38). The collected patient data will also enhance understanding of risk factors and protective factors for adverse outcomes that may provide targets for health care innovation. At last, structured multidisciplinary follow-up will not only provide answers to scientific questions but may also support the generation of new research initiatives and easier implementation of research and intervention studies in follow-up practice. For example, since patients were admitted due to MIS-C, we collaborate in outcome research with six other PICUs in The Netherlands (39). A blueprint of our program has been implemented in these PICUs.

Helpful recommendations for successfully starting and managing PICU follow-up programs have recently been reviewed by Williams et al (11). Although our program is in line with most of these recommendations, structured multidisciplinary follow-up faces limitations and challenges. The implementation of new initiatives may come across cautious attitudes among health care professionals and patients and their parents and may pertain to the clinical usefulness of standardized outcome measures, which potentially has a negative impact on compliance and participation. Another challenge is that our program is labor intensive. Several measures have been taken to improve time-efficiency. The completion of questionnaires on physical and psychosocial functioning before the consultations may help prepare parents for the consultation, save professionals time in history taking, and help focus the consultation on the specific issues reported (21). Time-efficiency for patients, parents, and professionals is promoted by scheduling all consultations consecutively with the multidisciplinary team meeting scheduled after the last consultation. Clinician-reported outcomes are registered in discrete registrations and automatically appear in a standardized written report, which is sent to the general practitioner and other treating physicians. To facilitate health care evaluation sessions, all data from the electronic patient records collected during PICU admission and during follow-up are automatically visualized in a real-time available dashboard built in Microsoft PowerBI (eFig. 1, http://links.lww.com/PCC/C337; legend, http://links.lww.com/PCC/C336). Despite aforementioned measures to improve time-efficiency, our program remains labor intensive, and therefore the involved health care professionals receive dedicated time. The implementation of a new program also comes with technological challenges, including building questionnaires and registration forms in the electronic patient file for discrete registration and the building of dashboards to support the health care evaluation sessions. Furthermore, selection bias may occur as patients whose functioning is impaired may be more likely to participate. However, this risk is much lower compared with most cross-sectional studies, as a result of the prospective inclusion of a consecutive cohort in the structured multidisciplinary follow-up program. Finally, a considerable number of patients and parents did not complete all questionnaires sent out before their visit to the outpatient clinic, resulting in inefficient consultations and an incomplete and limited view on their functioning. In our experience, a dedicated team is essential to tackle these challenges.

Future work needs to address the cost-effectiveness of structured multidisciplinary follow-up. The recently published article by Williams et al (11) identified “lack of support,” including lack of availability of funding and lack of institutional or departmental support, as the most important barrier with respect to the development and maintenance of PICU follow-up programs. Prior to implementation of our structured multidisciplinary follow-up program, funding was provided by our hospital for the development and implementation of the program. After development of a national guideline on follow-up of PICU patients (18), our structured multidisciplinary follow-up care was acknowledged and reimbursed by insurance companies. Professionals involved in the follow-up programs have been able to attract external funding for scientific research in several of the outcomes targeted. Only a structured follow-up program will provide insights in potential deleterious consequences of PICU admission, thereby facilitating targeted follow-up and enabling us to know which possible complications we have to focus on during PICU admission in order to prevent these complications and thereby also minimize costs after PICU discharge.

In order to gain a generalizable understanding of sequelae after pediatric critical illness, standardization of outcome assessment is urgently needed. Core outcome set and instrument recommendations have recently been developed by the Pediatric Outcomes STudies after PICU Investigators of the Pediatric Acute Lung Injury and Sepsis Investigators Network and the *Eunice Kennedy Shriver* National Institute of Child Health and Human Development Collaborative Pediatric Critical Care Research Network (10, 40). Although our follow-up program was developed well before publication of this core outcome set, the outcomes and instruments of our program largely converge with the recommendations made. Our structured multidisciplinary PICU follow-up program serves as an example of how clinical care, health care evaluation, and scientific research can be integrated to continuously provide data-driven health care innovation. Depending on, among others, availability of financial resources, health care professionals, language appropriate questionnaires, electronic patient record, and infrastructure to allow structured data collection, other PICUs may adapt this program in order to be applicable in their country and hospital. A strength of our set-up is the flexibility that this program has with regard to outcome measurements. Possible adaptations may arise from, for example, the evaluation sessions, new insights on outcomes, new diseases such as MIS-C requiring monitoring, or new tools or new releases of outcome measurements. Even when adaptations are made, a solid structure and process is preserved in which outcome monitoring is used for the purpose of improvement of patient outcomes, health care evaluation, and scientific research.

## CONCLUSIONS

We described the process of successful development and implementation of a structured, yet dynamic, multidisciplinary follow-up program for patients and their parents after PICU admission. Structured follow-up and collection of outcome data within a structured multidisciplinary follow-up program can importantly contribute 1) to improve outcomes of individual patients by facilitating timely support and appropriate intervention, if required; 2) to improve the standard of care during and after PICU admission by providing insight in extent and severity of sequelae; and 3) to facilitate scientific research on outcome and prognosis of patients after PICU admission. Given the distinct heterogeneity of the PICU population and wide range of potential impairments in functioning, we propose that structured multidisciplinary follow-up is required for patients and their parents after PICU admission. The accumulating outcome data will ultimately provide better insight in disease- and treatment-specific patient outcomes, thereby facilitating targeted follow-up of high-risk children and allowing further tailoring of the content of follow-up offered to those domains of functioning threatened.

## ACKNOWLEDGMENTS

We thank Alice Karsten, Nanette Stevens, Mariska Möhlmann, Francis Hofman, Rowena Prins, Marja Witteman, Jan Wijnia, Lara Busscher, Sonja Visser, Emilio Moes, Tom Bergman, Hedy van Oers, and Mieke Ouwendijk. The Emma Children’s Hospital Amsterdam UMC Follow Me program consortium collaborators are as follows: Joost Rotteveel, MD, PhD, Amsterdam UMC, University of Amsterdam & Vrije Universiteit, Emma Children’s Hospital, Department of Pediatrics, Amsterdam Reproduction and Development Research Institute, Meibergdreef 9, Amsterdam, The Netherlands; E-mail: j.rotteveel@amsterdamumc.nl. Eric G. Haarman, MD, PhD, Amsterdam UMC, University of Amsterdam, Emma Children’s Hospital, Department of Pediatric Pulmonology and Allergy, Amsterdam Reproduction and Development & Infection and Immunity Research Institutes, Meibergdreef 9, Amsterdam, The Netherlands; E-mail: eg.haarman@amsterdamumc.nl. Kim J. Oostrom, PhD, Amsterdam UMC, University of Amsterdam & Vrije Universiteit, Emma Children’s Hospital, Department of Child & Adolescent Psychiatry and Psychosocial Care, Amsterdam Reproduction and Development Research Institute, Meibergdreef 9, Amsterdam, The Netherlands; E-mail: kj.oostrom@amsterdamumc.nl. Margreet J. van Zelm - van Eldik, Amsterdam UMC, University of Amsterdam & Vrije Universiteit, Emma Children’s Hospital, Department of Child & Adolescent Psychiatry and Psychosocial Care, Amsterdam Reproduction and Development Research Institute, Meibergdreef 9, Amsterdam, The Netherlands; E-mail: m.j.vanzelmvaneldik@amsterdamumc.nl. L.W. Ernest van Heurn, MD, PhD, Amsterdam UMC, University of Amsterdam & Vrije Universiteit, Emma Children’s Hospital, Department of Pediatric Surgery, Amsterdam Gastroenterology Endocrinology Metabolism & Amsterdam Reproduction and Development Research Institutes, Meibergdreef 9, Amsterdam, The Netherlands; E-mail: e.vanheurn@amsterdamumc.nl. Ramon R. Gorter, MD, PhD, Amsterdam UMC, University of Amsterdam & Vrije Universiteit, Emma Children’s Hospital, Department of Pediatric Surgery, Amsterdam Gastroenterology Endocrinology Metabolism & Amsterdam Reproduction and Development Research Institutes, Meibergdreef 9, Amsterdam, The Netherlands; E-mail: rr.gorter@amsterdamumc.nl. Joep P.M. Derikx, MD, PhD, Amsterdam UMC, University of Amsterdam & Vrije Universiteit, Emma Children’s Hospital, Department of Pediatric Surgery, Amsterdam Gastroenterology Endocrinology Metabolism & Amsterdam Reproduction and Development Research Institutes, Meibergdreef 9, Amsterdam, The Netherlands; E-mail: j.derikx@amsterdamumc.nl. Anton H. van Kaam, MD, PhD, Amsterdam UMC, University of Amsterdam, Emma Children’s Hospital, Department of Neonatology, Amsterdam Reproduction and Development Research Institute, Meibergdreef 9, Amsterdam, The Netherlands; E-mail: a.h.vankaam@amsterdamumc.nl. Aleid G. Leemhuis, MD, PhD, Amsterdam UMC, University of Amsterdam, Emma Children’s Hospital, Department of Neonatology, Amsterdam Reproduction and Development Research Institute, Meibergdreef 9, Amsterdam, The Netherlands; E-mail: a.g.leemhuis@amsterdamumc.nl. Nico A. Blom, MD, PhD, Amsterdam UMC, University of Amsterdam, Emma Children’s Hospital, Department of Pediatric Cardiology, Amsterdam Cardiovascular Sciences Research Institute, Meibergdreef 9, Amsterdam, The Netherlands; E-mail: n.a.blom@amsterdamumc.nl. Irene M. Kuipers, MD, PhD, Amsterdam UMC, University of Amsterdam, Emma Children’s Hospital, Department of Pediatric Cardiology, Amsterdam Public Health Research Institute, Meibergdreef 9, Amsterdam, The Netherlands; E-mail: i.m.kuipers@amsterdamumc.nl.

## Supplementary Material


