# Supplementary material for: Structured Multidisciplinary Follow-Up After Pediatric Intensive Care: A Model for Continuous Data-Driven Health Care Innovation
Source: Pediatr Crit Care Med. 2023 Feb 17;24(6):484–98. doi: 10.1097/PCC.0000000000003213 (PMC10226472; doi:10.1097/PCC.0000000000003213)
Supplement: Supplementary file 1 [file pcc-24-484-s001.docx]

| **eTable 1.** Overview of patient and parent reported online questionnaires | | |  |
| --- | --- | --- | --- |
| **Outcome measurements** | **Questionnaire** | **Informant (parent and/ or child) and ages (years)** | **License required** |
| ***Patient outcomes*** |  |  |  |
| Somatic functioning | ISAAC questionnaire (1)  Custom-made questionnaire assessing school functioning, medical care after PICU discharge, development, vision and hearing, diuresis, defecation, skin, pain | Parent report (0-17)  Patient report (12-17) | No |
| Demographic characteristics | Custom-made questionnaire assessing parental authority, parental marital status, country of birth of patient and parents, number of children, parental education and work situation | Parent report (0-17) | No |
| Perinatal history | Custom-made questionnaire assessing pregnancy and labor mother and first weeks of life | Parent report (0-17) | No |
| Behavioral and emotional functioning | SDQ (2,3) | Parent report (2-17)  Patient report (11-17) | Yes (4) |
| Post-traumatic stress | CRIES-13 (5-7) | Parent report (3-17)  Patient report (8-17) | No |
| Health-related Quality of Life | TAPQOL (8,9) | Parent report (0-1) | No |
|  | PedsQL (10) | Parent report (2-7)  Patient report (8-17) | No (only consent of the originator professor J.W. Varni) |
| ***Additional patient outcomes in case of neurocognitive screening*** |  |  |  |
| Medical history related to neurocognitive functioning | Custom-made questionnaire assessing general anesthesia, traumatic brain injury, learning disability, neurological disability, psychiatric disability, language and/or speech disorder, developmental coordination disorder, intellectual disability, physical functioning, self-care, social functioning, cognitive abilities, attention, memory, executive functions, visual-spatial skills, motor functioning, school functioning | Parent report (6-17) | No |
| Behavior and emotional functioning | SWAN (11) | Parent report (6-17) | No |
| ***Parent outcomes*** |  |  |  |
| Parental post-traumatic stress | *Until February 2021:* SRS-PTSD (12)  *Since February 2021:* PCL-5 (13) | Parent report (0-17) | SRS-PTSD: No  PCL-5: No (free of charge after registration at Center 45: https://www.centrum45.nl/) |
| Parental distress | DT-P (14) | Parent report (0-17) | No |
| Parental anxiety and depression | *Until February 2021:* HADS (15,16)  *Since February 2021:* PROMIS-CAT Anxiety and Depression (17,18) | Parent report (0-17) | HADS: Yes  PROMIS-CAT Anxiety and Depression: Yes (19) |
| Note: CRIES-13 = Children’s Revised Impact of Event Scale; DT-P = Distress Thermometer for Parents; HADS = Hospital Anxiety and Depression Scale; ISAAC = International Study of Asthma and Allergies in Childhood; PCL-5 = PTSD Checklist for DSM-V; PedsQL = Pediatric Quality of Life Inventory; PROMIS-CAT = Parental-Reported Outcomes Measurement Information System Computer Adaptive Test Anxiety and Depression; SDQ = Strengths and Difficulties Questionnaire; SRS-PTSD = Self-Rating Scale for Posttraumatic Stress Disorders; TAPQOL = TNO-AZL Preschool Children Quality of Life Questionnaire; SWAN = Strengths and Weaknesses of Attention-Deficit/Hyperactivity Disorder Symptoms and Normal Behavior Scale. | | | |

| **eTable 2.** Overview of the clinician-reported outcomes | | | |
| --- | --- | --- | --- |
| **Involved health care professionals** | **Key references underpinning the importance of involved health care professionals and assessed outcomes** | **Clinician-reported outcomes** | **Details on** **clinician-reported outcomes** |
| ***Pediatric intensivist*** | (20-26) | History taking | Care after PICU discharge; withdrawal symptoms; eating; sleeping; diuresis; defecation; respiratory and cardiac complaints; psychomotor development. |
|  |  | Physical examination | General impression; head; thorax; airway; respiration; circulation; abdomen; skin; extremities; signs for post-thrombotic syndrome after central venous catheter. |
|  |  | Diagnostics for kidney function* | Blood: creatinine, ureum and cystatine C.  Urine: proteinuria (27-29) and microalbuminuria.(25)  Blood pressure: measured 3 times after 3 minutes of rest by the oscillometric Mindray VS-600 with the lowest value obtained being used to evaluate hypertension.(30) |
| ***Pediatric psychologist*** | (31-35) | Clinical interview with patient and parents | Health-related quality of life; behavior and emotional functioning; post-traumatic stress; parental post-traumatic stress; parental distress; parental anxiety and depression. |
| ***Pediatric pulmonologist*** | (24,36-38) | History taking | Voice; stridor; cyanosis; dyspnea; tachypnea; airway obstruction; exercise tolerance; coughing; breathing; airway infections. |
|  |  | Physical examination | Head; thorax; respiration; circulation; abdomen; extremities. |
|  |  | Lung function by spirometry | Spirometry before and after administration of short-acting-βs-mimetic with the calibrated spirometer (Vyntus SPIRO, Vyaire Medical, Inc). Measurements are performed according to the guideline of the American Thoracic Society and the European Respiratory Society.(39) The forced expiratory volume in 1 second (FEV1), forced vital capacity (FVC), maximal mid-expiratory flow at 25–75% of FVC (MMEF 75/25) and the Tiffeneau index (FEV1/FVC) are assessed and percentages of predicted values are based on the Global Lung Function Initiative standards.(40) |
| ***Pediatric cardiologist*** | (41) | History taking | Exercise tolerance; tachypnea; cyanosis; dyspnea; arrhythmias. |
|  |  | Physical examination | Respiration; circulation. |
|  |  | Electrocardiogram | General electrocardiogram. |
|  |  | Echocardiogram ** | 1) Biventricular systolic function: left ventricle (LV): LVSF (%), LVEF (%)(triplane en 4D), MAPSE (mm), global longitudinal LV strain (%) (2chamber/3chamber/4chamber), TDI (S' LV free wall en S' septum (cm/s)). Right ventricle (RV): TAPSE (mm), global longitudinal strain (%) (RV free wall), TDI (S' RV free wall (cm/s)).  2) Dimensions left and right ventricle (LVED (mm), LVES (mm), septal, posterior wall, right ventricular wall thickness (mm)).  3) Valve function: gradient (mmHg) over inflow and outflow valves, insufficiency.  4) Anatomy/shunts. |
| ***Pediatric neurologist*** | (20,21) | History taking | Epileptic attacks; headache; sensibility; motor skills. |
|  |  | Neurologic physical examination | Speech; assessment of cranial nerves; motor skills; coordination; sensibility; reflexes; gait pattern. |
| ***Pediatric neuropsychologist*** | (35,42,43) | Neurocognitive screening | Intelligence: WISC-V-NL short form.(44)  Neurocognitive computerized test battery (Emma Toolbox). This test-battery measures a broad range of key neurocognitive domains and contains a composition of child-friendly tests based on well-known neuroscientific paradigms with established validity and reliability, i.e. Attention Network Test,(45) Location Learning Test, (46) Rey Auditory Verbal Learning Test, (47) Klingberg task, (48) Digit Span task, (49) and Track & Trace task.(50) |
| Note: *Diagnostics for kidney function in case of PICU admission >48 hours and acute kidney injury during PICU admission (i.e. creatinine elevation >25% and/or diuresis <0.5ml/kg/hour for >8 hours) without known kidney disease. ** Abbreviations echocardiogram: LV: left ventricle, LVEF: left ventricular ejection fraction, LVSF: left ventricular shortening fraction, MAPSE: mitral annular plane systolic excursion, TAPSE: tricuspid annular plane systolic excursion, RV: right ventricle, TDI: tissue Doppler imaging. WISC-V-NL short form = short version of the Wechsler Intelligence Scale for Children, fifth version in Dutch. | | | |

**eFigure 1.** Example of one of the dashboards used for health care evaluation. The extracted data are visualized in a real time available dashboard built in Microsoft PowerBI.
Note: Each outcome of interest is covered by one or more main items. Some of these items offer follow-up items that require completion depending on the response chosen on the main item(s). The two graphs on the left (dark green, dark blue en grey colors) pertain to main items, while those on the right pertain to follow-up items.

**REFERENCES**

1. Asher MI, Keil U, Anderson HR, Beasley R, Crane J, Martinez F, et al. International Study of Asthma and Allergies in Childhood (ISAAC): rationale and methods. *Eur Respir J* 1995; **8**(3): 483-91.

2. Goodman R, Meltzer H, Bailey V. The Strengths and Difficulties Questionnaire: a pilot study on the validity of the self-report version. *Eur Child Adolesc Psychiatry* 1998; **7**(3): 125-30.

3. van Widenfelt BM, Goedhart AW, Treffers PD, Goodman R. Dutch version of the Strengths and Difficulties Questionnaire (SDQ). *Eur Child Adolesc Psychiatry* 2003; **12**(6): 281-9.

4. Youth in Mind. Accessible at: <https://youthinmind.com/products-and-services/sdq/>

5. Children’s Revised Impact of Event Scale (CRIES-13). Children and War Foundation. Available at: <http://www.childrenandwar.org>; 2005.

6. Verlinden E, van Laar YL, van Meijel EP, Opmeer BC, Beer R, de Roos C, et al. A parental tool to screen for posttraumatic stress in children: first psychometric results. *J Trauma Stress* 2014; **27**(4): 492-5.

7. Verlinden E, van Meijel EP, Opmeer BC, Beer R, de Roos C, Bicanic IA, et al. Characteristics of the Children's Revised Impact of Event Scale in a clinically referred Dutch sample. *J Trauma Stress* 2014; **27**(3): 338-44.

8. Fekkes M, Theunissen NC, Brugman E, Veen S, Verrips EG, Koopman HM, et al. Development and psychometric evaluation of the TAPQOL: a health-related quality of life instrument for 1-5-year-old children. *Qual Life Res* 2000; **9**(8): 961-72.

9. Bunge EM, Essink-Bot ML, Kobussen MP, van Suijlekom-Smit LW, Moll HA, Raat H. Reliability and validity of health status measurement by the TAPQOL. *Arch Dis Child* 2005; **90**(4): 351-8.

10. Varni JW, Limbers CA. The pediatric quality of life inventory: measuring pediatric health-related quality of life from the perspective of children and their parents. *Pediatr Clin North Am* 2009; **56**(4): 843-63.

11. Swanson JM, Schuck S, Porter MM, Carlson C, Hartman CA, Sergeant JA, et al. Categorical and Dimensional Definitions and Evaluations of Symptoms of ADHD: History of the SNAP and the SWAN Rating Scales. *Int J Educ Psychol Assess* 2012; **10**(1): 51-70.

12. Carlier IV, Lamberts RD, Van Uchelen AJ, Gersons BP. Clinical utility of a brief diagnostic test for posttraumatic stress disorder. *Psychosom Med* 1998; **60**(1): 42-7.

13. Blevins CA, Weathers FW, Davis MT, Witte TK, Domino JL. The Posttraumatic Stress Disorder Checklist for DSM-5 (PCL-5): Development and Initial Psychometric Evaluation. *J Trauma Stress* 2015; **28**(6): 489-98.

14. Haverman L, van Oers HA, Limperg PF, Houtzager BA, Huisman J, Darlington AS, et al. Development and validation of the distress thermometer for parents of a chronically ill child. *J Pediatr* 2013; **163**(4): 1140-6 e2.

15. Bjelland I, Dahl AA, Haug TT, Neckelmann D. The validity of the Hospital Anxiety and Depression Scale. An updated literature review. *J Psychosom Res* 2002; **52**(2): 69-77.

16. Van Hemert AM, Ormel J. Dutch version of the Hospital Anxiety and Depression scale (HADS) [Nederlandse versie van de Hospital Anxiety and Depression scale (HADS)]. Rijksuniversiteit Groningen; 1996.

17. Flens G, Smits N, Terwee CB, Dekker J, Huijbrechts I, Spinhoven P, et al. Development of a Computerized Adaptive Test for Anxiety Based on the Dutch-Flemish Version of the PROMIS Item Bank. *Assessment* 2019; **26**(7): 1362-74.

18. Flens G, Smits N, Terwee CB, Dekker J, Huijbrechts I, de Beurs E. Development of a Computer Adaptive Test for Depression Based on the Dutch-Flemish Version of the PROMIS Item Bank. *Eval Health Prof* 2017; **40**(1): 79-105.

19. Dutch-Flemish PROMIS National Center. Available at: <http://www.dutchflemishpromis.nl/>.

20. Knoester H, Bronner MB, Bos AP. Surviving pediatric intensive care: physical outcome after 3 months. *Intensive Care Med* 2008; **34**(6): 1076-82.

21. Knoester H, Grootenhuis MA, Bos AP. Outcome of paediatric intensive care survivors. *Eur J Pediatr* 2007; **166**(11): 1119-28.

22. Sol JJ, Knoester H, de Neef M, Smets AM, Betlem A, van Ommen CH. Chronic Complications After Femoral Central Venous Catheter-related Thrombosis in Critically Ill Children. *J Pediatr Hematol Oncol* 2015; **37**(6): 462-7.

23. Schweiger C, Marostica PJ, Smith MM, Manica D, Carvalho PR, Kuhl G. Incidence of post-intubation subglottic stenosis in children: prospective study. *J Laryngol Otol* 2013; **127**(4): 399-403.

24. Zomer-Kooijker K, van der Ent CK, Ermers MJ, Uiterwaal CS, Rovers MM, Bont LJ. Increased risk of wheeze and decreased lung function after respiratory syncytial virus infection. *PLoS One* 2014; **9**(1): e87162.

25. Mammen C, Al Abbas A, Skippen P, Nadel H, Levine D, Collet JP, et al. Long-term risk of CKD in children surviving episodes of acute kidney injury in the intensive care unit: a prospective cohort study. *American journal of kidney diseases : the official journal of the National Kidney Foundation* 2012; **59**(4): 523-30.

26. Gupta S, Sengar GS, Meti PK, Lahoti A, Beniwal M, Kumawat M. Acute kidney injury in Pediatric Intensive Care Unit: Incidence, risk factors, and outcome. *Indian journal of critical care medicine : peer-reviewed, official publication of Indian Society of Critical Care Medicine* 2016; **20**(9): 526-9.

27. Fathallah-Shaykh SA, Flynn JT, Pierce CB, Abraham AG, Blydt-Hansen TD, Massengill SF, et al. Progression of pediatric CKD of nonglomerular origin in the CKiD cohort. *Clinical journal of the American Society of Nephrology : CJASN* 2015; **10**(4): 571-7.

28. Warady BA, Abraham AG, Schwartz GJ, Wong CS, Munoz A, Betoko A, et al. Predictors of Rapid Progression of Glomerular and Nonglomerular Kidney Disease in Children and Adolescents: The Chronic Kidney Disease in Children (CKiD) Cohort. *American journal of kidney diseases : the official journal of the National Kidney Foundation* 2015; **65**(6): 878-88.

29. van der Heijden AJ, van Wijk JAE. Werkboek kindernefrologie. Chapter 5, Table 5-2. 2010.

30. Flynn JT, Kaelber DC, Baker-Smith CM, Blowey D, Carroll AE, Daniels SR, et al. Clinical Practice Guideline for Screening and Management of High Blood Pressure in Children and Adolescents. *Pediatrics* 2017; **140**(3).

31. Cunha F, Mota T, Teixeira-Pinto A, Carvalho L, Estrada J, Marques A, et al. Factors associated with health-related quality of life changes in survivors to pediatric intensive care. *Pediatr Crit Care Med* 2013; **14**(1): e8-15.

32. Bronner MB, Knoester H, Bos AP, Last BF, Grootenhuis MA. Posttraumatic stress disorder (PTSD) in children after paediatric intensive care treatment compared to children who survived a major fire disaster. *Child Adolesc Psychiatry Ment Health* 2008; **2**(1): 9.

33. Bronner MB, Peek N, Knoester H, Bos AP, Last BF, Grootenhuis MA. Course and predictors of posttraumatic stress disorder in parents after pediatric intensive care treatment of their child. *J Pediatr Psychol* 2010; **35**(9): 966-74.

34. Price J, Kassam-Adams N, Alderfer MA, Christofferson J, Kazak AE. Systematic Review: A Reevaluation and Update of the Integrative (Trajectory) Model of Pediatric Medical Traumatic Stress. *J Pediatr Psychol* 2016; **41**(1): 86-97.

35. Bronner MB, Knoester H, Sol JJ, Bos AP, Heymans HS, Grootenhuis MA. An explorative study on quality of life and psychological and cognitive function in pediatric survivors of septic shock. *Pediatr Crit Care Med* 2009; **10**(6): 636-42.

36. Miller MR, Hankinson J, Brusasco V, Burgos F, Casaburi R, Coates A, et al. Standardisation of spirometry. *Eur Respir J* 2005; **26**(2): 319-38.

37. Dahlem P, van Aalderen WM, Hamaker ME, Dijkgraaf MG, Bos AP. Incidence and short-term outcome of acute lung injury in mechanically ventilated children. *Eur Respir J* 2003; **22**(6): 980-5.

38. Plötz FB, van Vught H, Uiterwaal CS, Riedijk M, van der Ent CK. Exercise-induced oxygen desaturation as a late complication of meningococcal septic shock syndrome. *Jama* 2001; **285**(3): 293-4.

39. Miller MR, Crapo R, Hankinson J, Brusasco V, Burgos F, Casaburi R, et al. General considerations for lung function testing. *Eur Respir J* 2005; **26**(1): 153-61.

40. Cooper BG, Stocks J, Hall GL, Culver B, Steenbruggen I, Carter KW, et al. The Global Lung Function Initiative (GLI) Network: bringing the world's respiratory reference values together. *Breathe (Sheff)* 2017; **13**(3): e56-e64.

41. Knoester H, Sol JJ, Ramsodit P, Kuipers IM, Clur SA, Bos AP. Cardiac function in pediatric septic shock survivors. *Arch Pediatr Adolesc Med* 2008; **162**(12): 1164-8.

42. Fiser DH, Long N, Roberson PK, Hefley G, Zolten K, Brodie-Fowler M. Relationship of pediatric overall performance category and pediatric cerebral performance category scores at pediatric intensive care unit discharge with outcome measures collected at hospital discharge and 1- and 6-month follow-up assessments. *Crit Care Med* 2000; **28**(7): 2616-20.

43. Elison S, Shears D, Nadel S, Sahakian B, Garralda ME. Neuropsychological function in children following admission to paediatric intensive care: a pilot investigation. *Intensive Care Med* 2008; **34**(7): 1289-93.

44. Wechsler D. Wechsler Intelligence Scale for Children, Dutch version (5th ed.) (WISC-V). Amsterdam: Pearson Benelux B.V.; 2018.

45. Fan J, McCandliss BD, Sommer T, Raz A, Posner MI. Testing the efficiency and independence of attentional networks. *J Cogn Neurosci* 2002; **14**(3): 340-7.

46. Bucks RS, Willison JR. Development and validation of the location learning test (LLT): A test of visuo-spatial learning designed for use with older adults and in dementia. The Clinical Neuropsychologist; 1997. p. 273-86.

47. Kingma A, van den Burg W. Three parallel versions of the Rey Auditory Verbal Learning Test for children Dutch version: instructions & normative data [Drie parallelversies van de 15-woordentest voor kinderen: handleiding & normering]. Stichting Kinderneuropsychologie Noord Nederland 2005.

48. Nutley SB, Söderqvist S, Bryde S, Humphreys K, Klingberg T. Measuring working memory capacity with greater precision in the lower capacity ranges. *Dev Neuropsychol* 2010; **35**(1): 81-95.

49. Wechsler D. Wechsler Intelligence Scale for Children (3rd ed.) (WISC-III): Manual. San Antonio, TX: The Psychological Corporation.; 1991.

50. De Kieviet JF, Stoof CJ, Geldof CJ, Smits N, Piek JP, Lafeber HN, et al. The crucial role of the predictability of motor response in visuomotor deficits in very preterm children at school age. *Dev Med Child Neurol* 2013; **55**(7): 624-30.
