# Supplementary figures and images for: Structured Multidisciplinary Follow-Up After Pediatric Intensive Care: A Model for Continuous Data-Driven Health Care Innovation
Source: Pediatr Crit Care Med. 2023 Feb 17;24(6):484–98. doi: 10.1097/PCC.0000000000003213 (PMC10226472; doi:10.1097/PCC.0000000000003213)

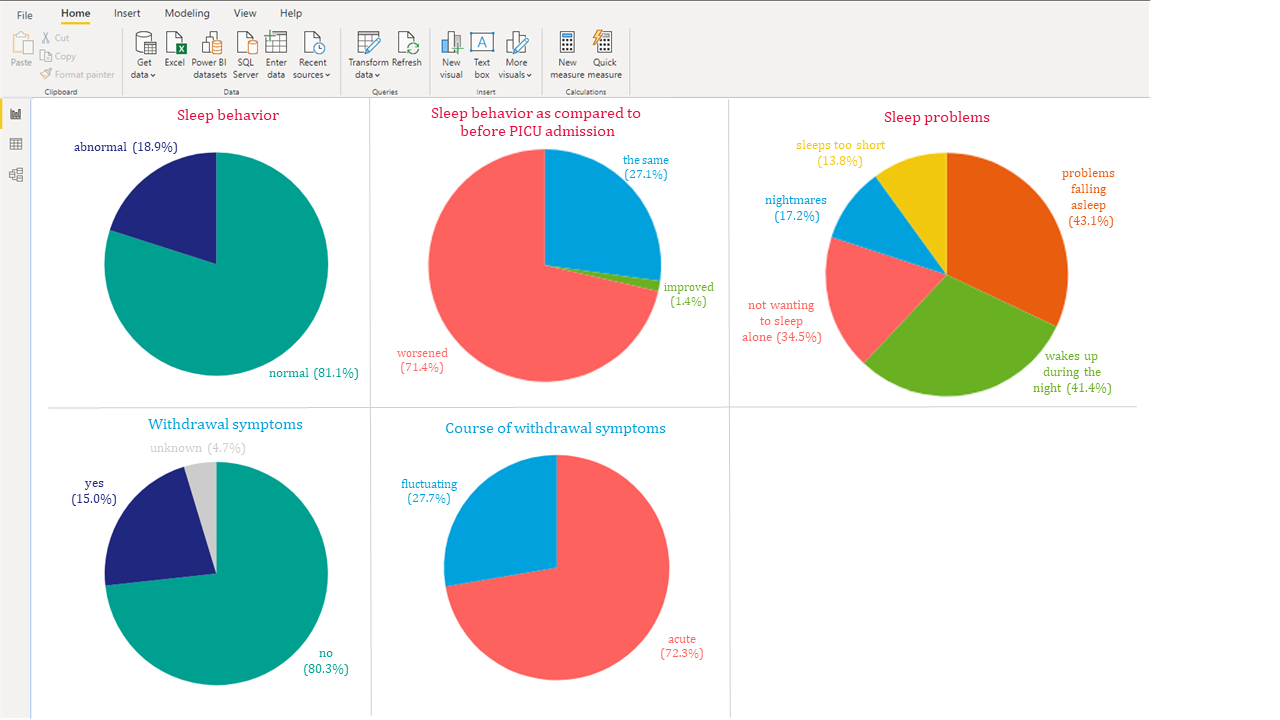

Supplement: Supplementary file 2 [file pcc-24-484-s002.tif]
